# Excitation and contraction of cardiac muscle and coronary arteries of brain‐dead pigs

**DOI:** 10.1096/fba.2022-00104

**Published:** 2022-12-26

**Authors:** Per Arlock, Mei Li, Benjamin Davis, Cecilia Lövdahl, Qiuming Liao, Trygve Sjöberg, Awahan Rahman, Björn Wohlfart, Stig Steen, Anders Arner

**Affiliations:** ^1^ Department of Clinical Sciences Lund, Lund University Lund Sweden; ^2^ Department of Physiology and Pharmacology Karolinska Institutet Stockholm Sweden

**Keywords:** action potential, Ca^2+^ imaging, cardiac transplantation, coronary artery, L‐type channels

## Abstract

Excitability and contraction of cardiac muscle from brain‐dead donors critically influence the success of heart transplantation. Membrane physiology, Ca^2+^‐handling, and force production of cardiac muscle and the contractile properties of coronary arteries were studied in hearts of brain‐dead pigs. Cardiac muscle and vascular function after 12 h brain death (decapitation between C2 and C3) were compared with properties of fresh tissue. In both isolated cardiomyocytes (whole‐cell patch clamp) and trabecular muscle (conventional microelectrodes), action potential duration was shorter in brain dead, compared to controls. Cellular shortening and Ca^2+^ transients were attenuated in the brain dead, and linked to lower mRNA expression of L‐type calcium channels and a slightly lower I_Ca_,_L_, current, as well as to a lower expression of phospholamban. The current–voltage relationship and the current above the equilibrium potential of the inward K^+^ (I_K1_) channel were altered in the brain‐dead group, associated with lower mRNA expression of the Kir2.2 channel. Delayed K^+^ currents were detected (I_Kr_, I_Ks_) and were not different between groups. The transient outward K^+^ current (I_to_) was not observed in the pig heart. Coronary arteries exhibited increased contractility and sensitivity to the thromboxane analogue (U46619), and unaltered endothelial relaxation. In conclusion, brain death involves changes in cardiac cellular excitation which might lower contractility after transplantation. Changes in the inward rectifier K^+^ channel can be associated with an increased risk for arrhythmia. Increased reactivity of coronary arteries may lead to increased risk of vascular spasm, although endothelial relaxant function was well preserved.

## Graphical abstract

A large animal (porcine) model for 12‐hour brain death was used to examine properties of the donor heart. The model mimicked the characteristics of human brain death with catecholaminergic surge and arterial pressure drop. Isolated trabecular preparations and cells revealed shorter action potentials, lower Ca^2+^ transients and impaired shortening. Electrophysiology revealed lower L‐type currents and decreased inward rectifier K^+^ currents. Coronary arteries had increased sensitivity to thromboxane activation, but unaltered endothelial relaxation.

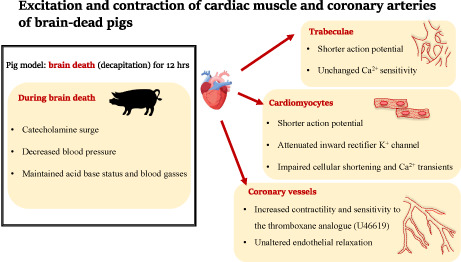

## 1 INTRODUCTION

Cardiac transplantation is a key technique in the treatment of end‐stage heart failure. Donor hearts are obtained from brain‐dead patients and should have well preserved functional properties to avoid post‐transplantation complications. The procedure must also include consideration of time aspects to enable adequate time for the clinical preparation prior to the transplantation, and for interaction with the patient's relatives. The brain death involves several potentially detrimental events (e.g., the hemodynamic alteration, catecholamine storm, other hormonal and electrolyte changes and the possibility of impaired cardiac perfusion), cf. Refs. [1, 2] and significant clinical efforts are made to avoid injury to organs during a period of brain death.^3^ Current donor management strategies are focused on normalization of hormone levels and on maintaining adequate circulation.^3^, ^4^, ^5^ Still, the function of the heart of the brain‐dead individual, including factors affecting perfusion, contractility and excitability of the myocardium, is not fully understood. A limited number of experiments in brain‐death animal models exist, and, for example, studies of coronary artery function have shown upregulated responses to serotonin and varying results regarding endothelial function.^6^, ^7^ Also, studies of excitation‐contraction in the myocardium are few. Experiments on 3‐h brain death in pigs have suggested that no major changes in epicardial action potential occur.^8^ Further characterization of coronary vessels and myocardium of the brain‐dead heart is needed.

Pig hearts have an overall anatomy and function closely resembling those of humans, constituting the basis for several clinically relevant studies using this animal model. Recently, the pig heart has also been proposed and applied, as a donor in xenotransplantation.^9^, ^10^, ^11^ In this context, information on the general properties of pig cardiac muscle is now needed. We have initiated a study of cardiac function in a porcine model for cardiac transplantation. In this report, we have compared the in vitro function of cardiac muscle and coronary arteries in fresh hearts and in hearts of brain‐dead animals. The pig model of brain death exhibits the main characteristics of human brain death, with catecholamine storm and lowering of blood pressure.^12^ We aimed at identifying cardiac alterations during a 12 h brain‐death period with minimal treatment efforts, which consisted only of basic intravenous fluid and electrolyte support. This would, for example, correspond to a clinical situation where brain herniation occurs in a patient during off‐hours, making cardiac donation impossible in most cases. An important aspect is if these hearts could be rescued by treatment and our aim was therefore to identify factors in the heart that are changed by the long‐term brain death period, as a basis for possible recovery initiatives. The results can also have a bearing on understanding of cardiac changes occurring earlier during a period of brain death. In vitro studies of isolated cardiac muscle and coronary arteries were applied to address the question: How does a period of brain death affect the membrane properties, calcium handling and contractility of cardiomyocytes and the contractile and endothelial function of the coronary arteries?

## 2 METHODS

### 2.1 Animal model of brain death

A pig model of acute total brain and brain stem death by decapitation has been described,^5^, ^12^ and was used in the present study. Swedish domestic pigs of either sex with a mean weight of 48 kg (40–62 kg) were used. All experiments were approved by the local animal ethical committee (Lund/Malmö M233‐09, M389‐12 and M174‐15). The animals were divided into one control group and one brain‐dead group. In the control group, the harvesting of the heart was done about 60 minutes after induction of anesthesia. In the brain‐dead group, the harvesting was done 12 h after brain death. Anesthesia was induced with one dose of intramuscular ketamine (30 mg/kg). Before tracheotomy, sodium thiopental (5–8 mg/kg) and atropine (0.02 mg/kg) were given intravenously. During the operative procedures and the period of brain death, anesthesia (Ketamine 200 mg/h, Midazolam 6 mg/h) and muscle relaxation (Rocuronium 40 mg/h) were continued. A tracheal tube (7.0 mm internal diameter) was inserted via a tracheotomy. The tube was connected to a Servo Ventilator 300 (Siemens, Solna, Sweden), and normoventilation was used (8 ml/kg body weight, 20 breaths/min and a PEEP of 5 cm H_2_O (=0.49 kPa)). Catheters for blood pressure measurements were placed in the intrathoracic ascending aorta and the right atrium. Their position was confirmed at autopsy after the experiment. Blood pressure and ECG signals were sampled continuously to a computer during the experiment. Brain death was induced by ligating all blood vessels and dividing the muscles in the neck. The *columna vertebralis* was divided between the second and third vertebra, thus giving a decapitation, totally removing the whole brain and brainstem. All animals were given intravenous Ringer‐Glucose 3 ml/kg body weight per hour throughout the experiment without further treatment. Samples for analysis of blood gasses were taken, before induction of brain death and after 6 and 12 h. For harvesting of the heart, the chest was opened by sternotomy and the heart was visualized. Aorta was cross clamped distal to a cannula placed in *aorta ascendens* where one liter of cold (4°C) Plegisol® cardioplegic solution (Hospira Inc., Lake Forrest, Il) was infused into the coronary arteries. The right atrium was opened at the beginning of the infusion to enable outflow of blood. The harvested hearts were immediately immersed in cold (4°C) Plegisol® solution in a cooling box surrounded with ice, as in a clinical setting. The hearts were transported by air freight from Lund University to Karolinska Institutet, Stockholm. The transport took 4 h, and the temperature was kept constant.

After the heart had arrived at the Stockholm laboratory, it was kept in cold Plegisol® cardioplegic solution during tissue preparations (less than 15–30 min). Ventricular myocytes were isolated and, in parallel, coronary arteries were dissected for in vitro mechanical experiments and trabeculae prepared from the right ventricle for contractility measurements and electrophysiological recordings. Samples were also rapidly frozen in liquid N_2_ for mRNA analysis using quantitative PCR.

### 2.2 Isolation of ventricular myocytes

Ventricular myocytes were enzymatically isolated as described previously^13^, ^14^ from a wedge of the left ventricular free wall, supplied by the left anterior descending coronary artery. The cells were examined using patch clamp and Ca^2+^ imaging techniques.

### 2.3 Whole‐cell patch clamp

The whole‐cell variant of the patch‐clamp technique was used to record ionic currents and action potentials at 37°C.^14^ The resistance of the glass pipette was 1 to 5 MΩ when filled with an internal pipette solution (in mM: KCl 150, MgCl_2_ 5, Hepes 5, pH 7.2). The cell capacitance was determined using 10 mV pulses applied from a holding potential of −10 mV. The cells were superfused with a Tyrode solution. Voltage and current signals were stored on a personal computer with in house Linux‐Mandrake‐Comedi recording and analysis programs.

### 2.4 Membrane potential

Action potentials in cells in the Tyrode solution were elicited by 3 ms depolarizing pulses through the pipette. Measurements in trabeculae are described in section 2.7. The resting membrane potential, the action potential overshoot, and the action potential duration (APD) were evaluated. APD was analyzed by determining the time to reach 25%, 50%, and 90% repolarization (APD_25_, APD_50_, and APD_90_).

### 2.5 Calcium current (I_Ca_ )

A holding potential of −40 mV was used. I_Ca_ was elicited by imposing a series of 300 ms steps of different amplitude to a maximal potential of +40 mV (interval 5 s). The I_Ca_ amplitude was determined as the difference between peak current and the current level at the end of the pulse. The decay of I_Ca_ with time (t) was fitted by single or double exponential functions.

### 2.6 Potassium currents

The L‐type Ca^2+^ current (I_Ca_,_L_) was blocked with 5 μM Nifedipine. The inward rectifier K^+^ current (I_K1_) was determined using 200 ms voltage steps in the range −140 to −20 mV from a holding potential of −50 mV (interval, 3 s). The current was determined at the end of the 200 ms step. We also examined the presence of a transient outward K^+^ current (I_to_) by making 300 ms steps in the range −40 mV to +60 mV from a holding potential of −70 mV and a 10 ms pre‐pulse step to −45 mV (interval, 10 s). The delayed rectifier (I_K_) was examined using a 3 s step in the range −20 to +60 mV from a holding potential of −50 mV. The protocol was applied in 20 s intervals. The step current was analyzed as the increase in current developed at the end of the 3 s step. The slow component, I_Ks,_ was measured during blockade of I_Kr_ by 10 μM E‐4031 or 1 μM Dofetilide added to the superfusate, and I_Kr_ was measured during blockade of I_Ks_ by 30 μM Chromanol 293B.

### 2.7 Membrane potential and contraction of isolated trabecular muscle

Thin trabeculae from the right endocardium were mounted in a thermostated Plexiglass chamber and stretched at the start of the experiment to produce 90% maximum force. The preparations were paced at 1 s intervals through a pair of platinum electrodes using 3 ms rectangular current pulses with 20% above threshold amplitude. The cells in the tissue were impaled using conventional glass microelectrodes filled with 3 M KCl (resistance ~10 MΩ). The preparations were perfused with Krebs' solution at a temperature 36 ± 0.1°C. The signals were digitized at 10 kHz.

### 2.8 Quantitative reverse transcription PCR (RT‐qPCR) analysis

RNA was purified from the myocardial samples (RNeasy Kit, Qiagen) and transcribed to cDNA (QuantiTect Rev. Transcription Kit, Qiagen). Using qPCR, we quantified the expression of key membrane channel proteins and of Ca^2+^ translocation components (QuantiTect SYBR Green RT‐PCR Kit, Qiagen). The primers used for each gene are listed in Table 1. The relative gene expression in samples of hearts from control and brain‐dead animals was evaluated using the delta‐Ct method with GAPDH as housekeeping gene. It is noted that the mRNA expression does not necessarily reflect the level of corresponding protein, but is here related to functional changes.

**TABLE 1:** Primers used for RT‐qPCR analysis of pig cardiac samples

GENE/NCBI ID	mRNA	Forward	Reverse
GAPDH, ID: 396823	NM_001206359.1	CCTCAACGACCACTTCGTCA	GGCTCTTACTCCTTGGAGGC
CACNA1C, ID: 100518733	XM_013997911.1	GGACCTGAAAGGCTACCTGG	GTGGGCATGCTCATGTTTCG
KCNJ2, ID: 397293	NM_214151.1	CTTCGTCCTCTCGTGGCTAT	AAGCTGTTGACCTCGGACAC
KCNJ4, ID: 100523060	XM_003126023.5	TCCCGGAGACTCGTCGGA	TCTTCTTGACGAAGCGGTTGC
RyR2, ID: 396856	XM_013983211.1	CAGCGGAGGACAGTGCATTA	GGCCCCATCTTATGTGGCTT
SERCA2, ID: 396875	NM_213865.1	GAACGAGCAAATGCCTGCAA	ATCGATGTCCGGCTTGGTTT
PLN, ID: 397421	NM_214213.1	CAGCCAAGGCTGCCTAAAAGA	AGCAGAGCGAGTGAGGTATTG
ATP1B1, ID: 396898	NM_001001542.1	TGGCAAGCGTGACGAAGAT	GGAGCTTGCCGTAGTAAGGG

Abbreviations: ATP1B1, Na^+^/K^+^ transporting ATPase, beta‐1 subunit; CACNA1C, voltage‐dependent L‐type calcium channel subunit alpha‐1C (CaV1.2 channel); GAPDH, glyceraldehyde‐3‐phosphate dehydrogenase; KCNJ2, inwardly rectifying subfamily J, member 2 (Kir2.1 channel); KCNJ4, inwardly rectifying subfamily J, member 4 (Kir2.3 channel); PLN, phospholamban; RYR2, Ryanodine Receptor 2; SERCA2, Ca^2+^ transporting ATPase.

### 2.9 Measurement of intracellular [Ca^2+^]

Isolated cardiomyocytes were loaded at room temperature (22°C, 30 min) under dark conditions in 10 μM Fluo‐3/AM (Molecular Probes, Oregon USA) dissolved in a Ca^2+^ containing Krebs' solution and imaged using a confocal microscope (Zeiss LSM 510 Meta confocal microscope) in the line scan mode (488 nm excitation and 505 nm emission filters). Cells were placed in a flow‐through cuvette equipped with platinum stimulation electrodes and a glass window for fluorescence measurements. The cells were allowed to sediment, then superfused with the Krebs' solution, and electrically stimulated at 2 s intervals. The Ca^2+^ signal was evaluated as the fluorescence intensity (F) related to intensity in the relaxed state (F_0_). Shortening responses were evaluated as the maximal cellular shortening during the contractions. The mean value of measurements from approximately 10 cells was taken as representative for one heart.

### 2.10 Preparation and analysis of permeabilized trabecular muscle preparations

Skinned trabecular preparations were prepared using Triton‐X100.^15^ The fibers were stored in the relaxing solution with 50% glycerol for a maximum of 2–3 weeks at −20°C. The experiments were performed in solutions containing (mM): 30 imidazole, 5 MgATP, 12.5 phosphocreatine, 2 Mg^2+^, 5 EGTA, pH 7.0. The K_2_EGTA/KCaEGTA ratio was varied to obtain relaxing (pCa = −log [Ca^2+^] 9) and activating (pCa 4.5) solutions. 0.64 mg/ml creatine kinase was added. The composition of the solutions was calculated using a computer program similar to that previously described.^16^ The muscles were glued between two pins, one attached to an AME force transducer (SensoNor, Horten, Norway), the other to a micrometer screw, and stretched to a sarcomere length of 2.3 μm in relaxing solution. The fibers were first activated at pCa 4.5 to obtain a maximal Ca^2+^‐activated force, followed by relaxation, then activated at increasing [Ca^2+^] to determine the pCa‐force relationship.

### 2.11 Coronary arteries

The left anterior descending coronary artery (LAD) or the posterior descending artery (PDA) was isolated and placed in Krebs' solution at 4°C. The subepicardial artery tree was dissected along the main branch, and vessel segments with three different diameters were obtained: small (<500 μm), medium (500–1000 μm), and large (>1000 μm) (cf. Figure 6). Ring preparations were mounted in a multi‐wire myograph (610M, DMT, Denmark) with either stainless steel wires (40 μm, for smaller vessels) or pins (200 μm for medium and large vessels). From each heart, 2–4 preparations for each vessel size were analyzed, the average was taken as representative for the animal. The baths contained Krebs' solution. After 30 min equilibration, the muscles were repeatedly activated with high‐K^+^ solution (80 mM, 5 min) and relaxed in Krebs' solution (10 min). Vessel circumference was altered at the end of the relaxation period. For each circumference, the passive tension (end of relaxation period) and active tension (after 5 min of activation) were recorded. Vessel segment lengths were determined, and wall tension (force per wall length) was calculated. Subsequent experiments were performed at the optimal circumference (*L*_o_) for active force generation. The concentration‐tension dependence for the thromboxane A_2_‐receptor agonist U46619 (1 nM to 1 μM, Sigma‐Aldrich Sweden AB, Stockholm) was determined with the agonist added cumulatively in 5 min intervals. At the plateau of the contraction induced by 1 μM U46619, the vessels were challenged with substance P (10 nM) to determine the relaxation properties.

### 2.12 Composition of Solutions

Electrophysiological patch clamp recordings were made in a Tyrode solution containing (mM): NaCl 150, KCl 5.4, CaCl_2_ 2.0, MgCl_2_ 2.05, glucose 5.5 and Hepes 10, pH 7.4. Trabecular preparations were perfused with Krebs' solution at a temperature 36 ± 0.1°C containing (mM): NaCl 118, KCl 4.7, MgSO_4_ 1.2, CaCl_2_ 2.0, NaHCO_3_ 24.9, KH_2_PO_4_ 1.2, and glucose 10, gassed with 95/5% O_2_/CO_2_, pH 7.4. Coronary arteries were studied in Krebs' solution with (in mM) NaCl 123, KCl 4.7, KH_2_PO_4_ 1.2, MaCl_2_ 1.2, NaHCO_3_ 20, CaCl_2_ 2.5, glucose 5.5, gassed with 95/5% O_2_/CO_2_, pH 7.4 at 37°C.

### 2.13 Statistics

All values are presented as mean ± SEM. Unless otherwise stated, the n values refer to the number of animals. Statistical comparisons were made using Student's *t*‐test or, when appropriate, analysis of variance (ANOVA) with post hoc testing for multiple comparisons using the Holm‐Sidak method or repeated measured for dependent variables. Calculations and curve fitting were performed using SigmaPlot and SigmaStat for Windows (SPSS Science, Chicago, IL).

## 3 RESULTS

### 3.1 Data from anesthetized animals

**FIGURE 1:**
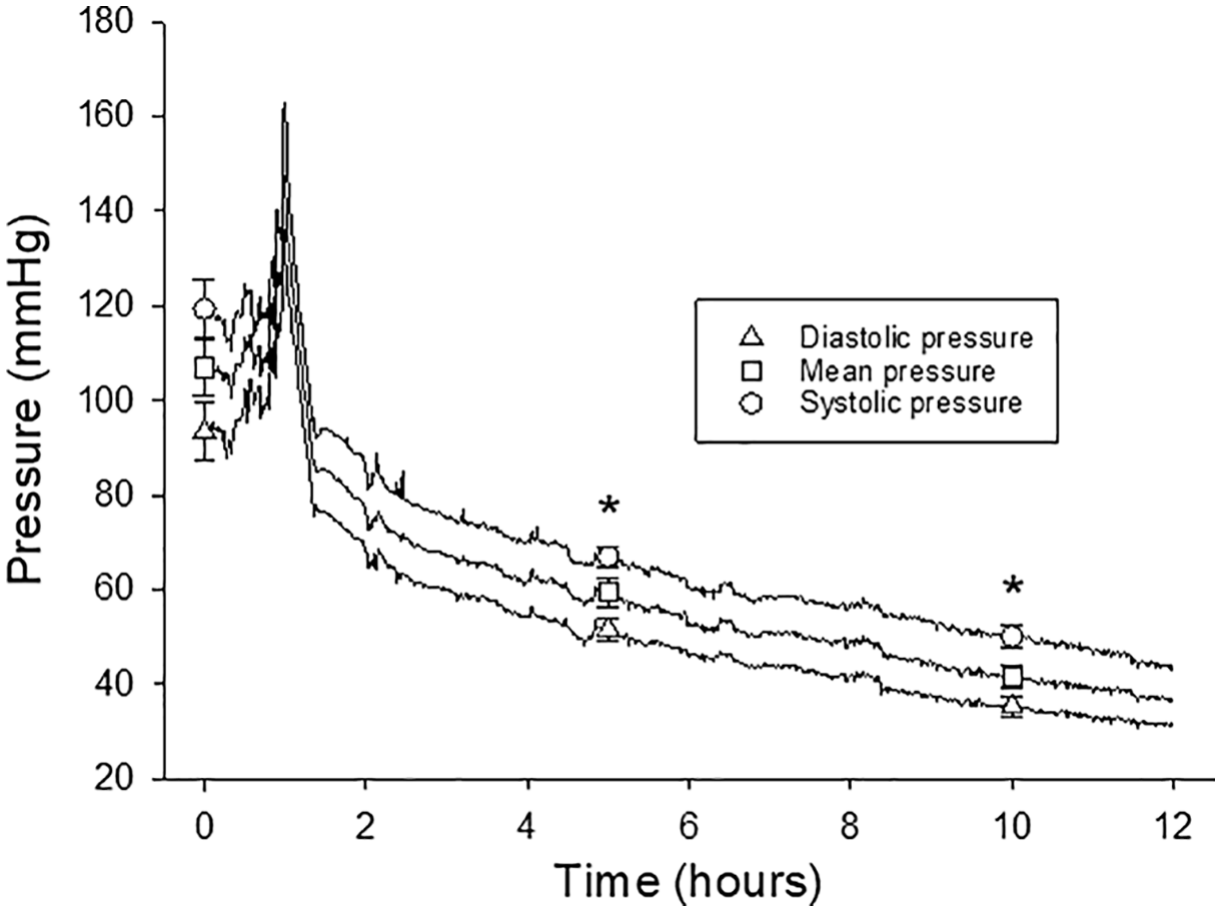
Aortic blood pressure in pigs during 12 h of brain death (*n* = 5). Mean values ± SEM are inserted at 5 h intervals. Note the initial pressure increase during the catecholamine storm following the removal of brain and brain stem. *p < 0.05 compared to the initial values for each pressure parameter.

Figure 1 shows mean values of blood pressure from brain‐dead animals. Following induction of brain death, a prominent transient pressure increase, reflecting the catecholamine surge, was observed. This was followed by a gradual decline in pressure during the 12 h as shown in the diagram. Urine production decreased and there was no urine production after 5 h, when mean aortic pressure was less than 60 mmHg. Blood gas and acid base status analyses (arterial PO_2_, PCO_2_, pH, base excess, and temperature) were performed before induction of brain death and at 6 and 12 h (F_i_O_2_ was 0.21). The values did not indicate major changes in metabolic status (**PO**_**2**_: Initial 15.2 ± 0.6, 6 h 15.6 ± 0.6, 12 h 16.2 ± 0.8 kPa; **PCO**_**2**_: Initial 4.2 ± 0.4, 6 h 3.7 ± 0.02, 12 h 3.7 ± 0.2 kPa; **pH**: Initial 7.47 ± 0.02, 6 h 7.50 ± 0.02, 12 h 7.48 ± 0.03 ‐log moles/L; **BE**: Initial −0.2 ± 1.5, 6 h 0.0 ± 1.6, 12 h 0.4 ± 1.6 mEq/L; **Temperature**: Initial 37.3 ± 0.3, 6 h 35.5 ± 0.4, 12 h 34.9 ± 0.3 °C).

### 3.2 Membrane potentials

**FIGURE 2:**
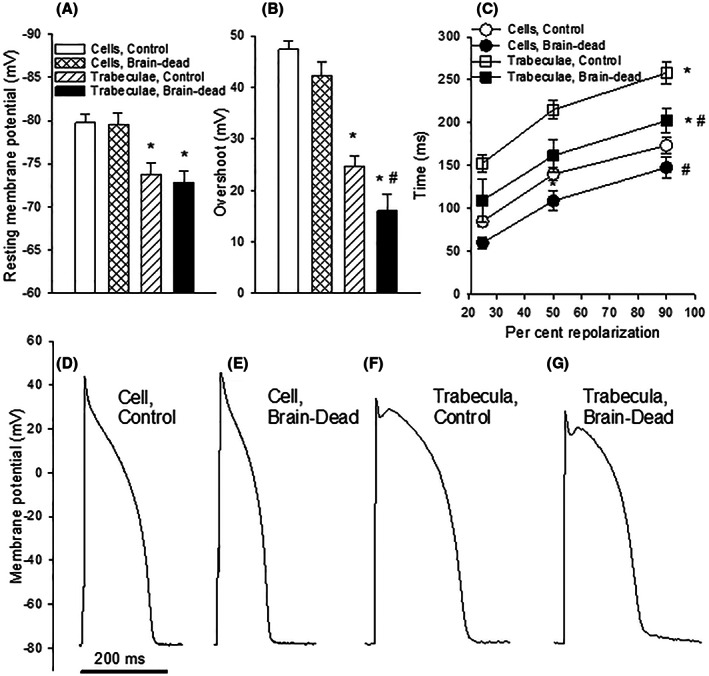
Resting membrane potential (panel A), action potential overshoot (panel B), time for repolarization (action potential duration, panel C) and original records of action potentials from cells (control: panel D; brain dead: panel E) and trabecular preparations (control: panel F; brain dead: panel G). Data in panels A–C were obtained from isolated cardiomyocytes (control hearts, *n* = 9–11; hearts of brain‐dead animals, *n* = 5–8) and from isolated trabecular muscle (control hearts, *n* = 10; hearts of brain‐dead animals, *n* = 5). *p < 0.05 compared to cells in the corresponding group. ^#^ *p* < 0.05 compared to the control group.

Membrane potentials were recorded from both isolated left ventricular cardiomyocytes and isometrically contracting trabecular muscle preparations (Figure 2). The resting membrane potential was less negative in the trabecular muscle preparations compared to that of the cells. The difference in membrane and action potentials between trabecular and cellular preparations most likely reflects influences by adjacent cells in the trabecular tissue. The recordings from these two different preparations show, however, that the resting membrane potential is similar in cardiac muscle in hearts from normal and brain‐dead animals. The overshoot of the action potential was lower in the trabecular preparations compared to that of the cells. The overshoot was slightly lower in preparations of brain‐dead animals compared that of controls in the cell experiments, and significantly lower in the trabecular experiments. The action potential duration was longer in the trabecular preparations compared to that of the cells. The action potentials of both cells and trabecular preparations were significantly shorter in the brain‐dead group compared to the controls (panel C, Figure 2).

### 3.3 Slow inward Ca^2+^ currents

**FIGURE 3:**
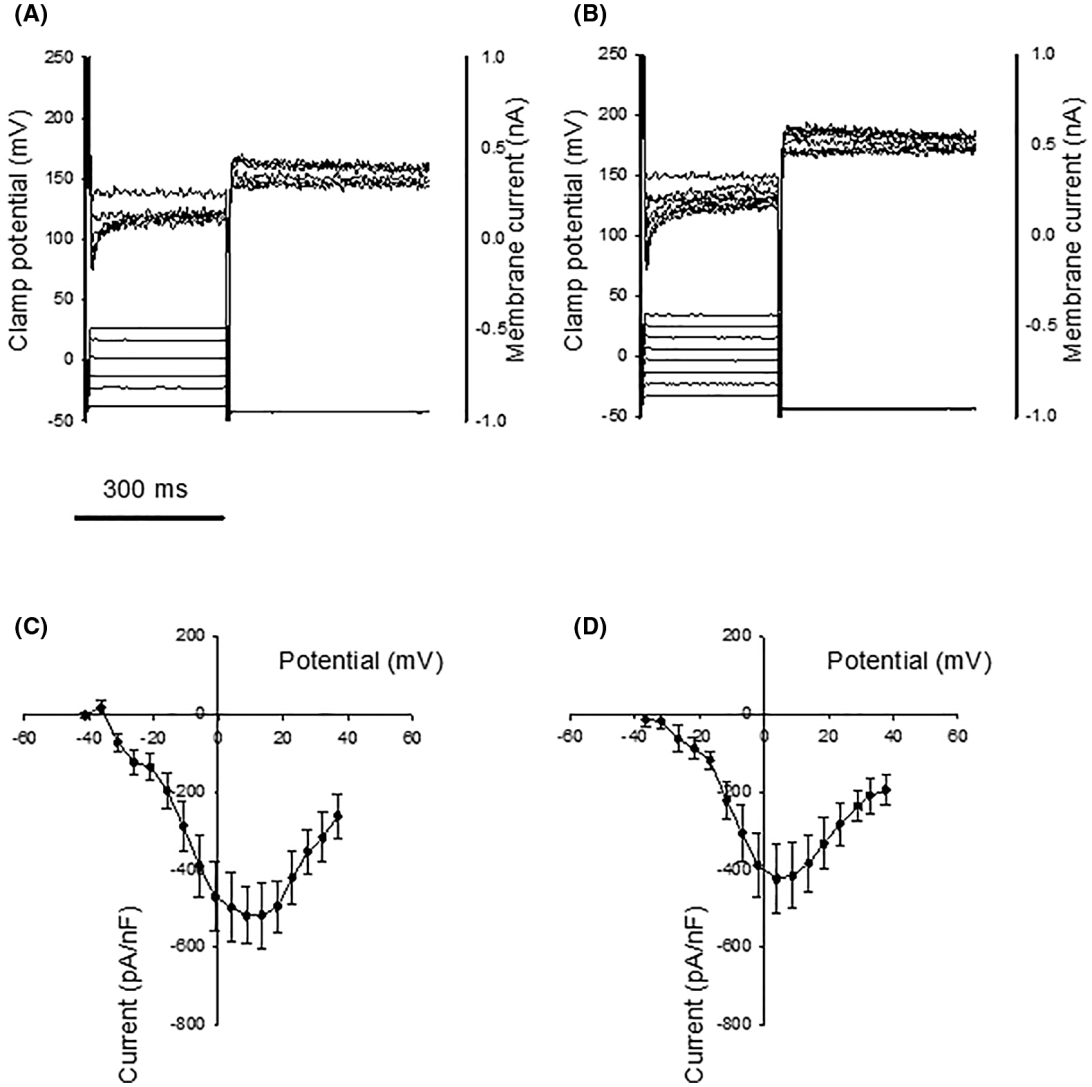
Original records of L‐type Ca^2+^ currents determined in cardiomyocytes dissociated from a control heart (panel A) and a heart from a brain‐dead animal (panel B). Top traces in each of these panels show the membrane currents in response to the voltage steps (lower traces) from a holding potential of −40 mV. Voltage–current relationships for L‐type Ca^2+^ channels determined in control hearts (10 cells from 8 animals, panel C), hearts from brain‐dead animals (6 cells from 3 animals, panel D). Peak current in response to voltage steps from a holding potential of −40 mV were analyzed. Currents were normalized to the cell capacitance.

The properties of the slow inward Ca^2+^ current (L‐type, I_Ca,L_) in isolated cardiomyocytes from control and brain‐dead animals are shown in Figure 3. The current–voltage relationships had similar shapes with a maximum inward current at slightly positive membrane potentials (controls: 9.5 ± 1.5 mV, *n* = 8; brain dead: 3.6 ± 2.4 mV, *n* = 7). The brain‐dead animals had slightly lower maximal currents (controls: 356 ± 45 pA, *n* = 22, brain dead 263 ± 48 pA, *n* = 7; when corrected for cell capacitance controls: 550 ± 93 pA/nF, *n* = 10 cells; brain dead 436 ± 90 pA/nF, *n* = 6). On average, the cell capacitance was similar in the two groups (controls: 544 ± 69 pF, *n* = 10; brain dead: 577 ± 41 pF, *n* = 6). The time constants for the inactivation of the L‐type Ca^2+^ current at the voltage step giving maximal current response were determined in a few cells in each group by fitting a mono‐exponential or a dual‐exponential function to the current trace. No major differences were detected (control heart: 81 ± 17 ms, *n* = 10; heart from brain animals: 114 ± 55 ms, *n* = 3). The electrophysiological measurements suggested a lower L‐type channel current in the brain‐dead group. We therefore determined the mRNA content for the α_1_ subunit of the voltage gated L‐type Ca^2+^ channel and found a significantly lower expression (Table 2), consistent with a lower Ca^2+^ current in the brain‐dead group.

**TABLE 2:** RT‐qPCR analysis and the mRNA expression in cardiac tissue from control (*n* = 4) and brain‐dead animals (*n* = 4)

GENE/Protein	Control	Brain dead
CACNA1C, CaV1.2, Ca^2+^ channel	0.0111 ± 0.0010	0.0059 ± 0.0008**
KCNJ2, Kir2.1, I_K1_ channel	0.0305 ± 0.0061	0.0358 ± 0.0004
KCNJ4, Kir2.3, I_K1_ channel	0.0041 ± 0.0005	0.0021 ± 0.0003*
RyR2, Ryanodine receptor	0.0058 ± 0.0017	0.0055 ± 0.0001
SERCA2, Sarcoplasmic reticulum Ca^2+^ ATPase	0.0366 ± 0.0095	0.0508 ± 0.0024
PLN, Phospholamban	1.1288 ± 0.2552	0.4233 ± 0.0675*
ATP1B1, Na+/K + ‐ATPase	0.0180 ± 0.0067	0.0190 ± 0.0034

* and ** indicate *p* < 0.05 and *p* < 0.01 compared to the control group.

### 3.4 Inward rectifier K^+^ currents (I_K1_ )

**FIGURE 4:**
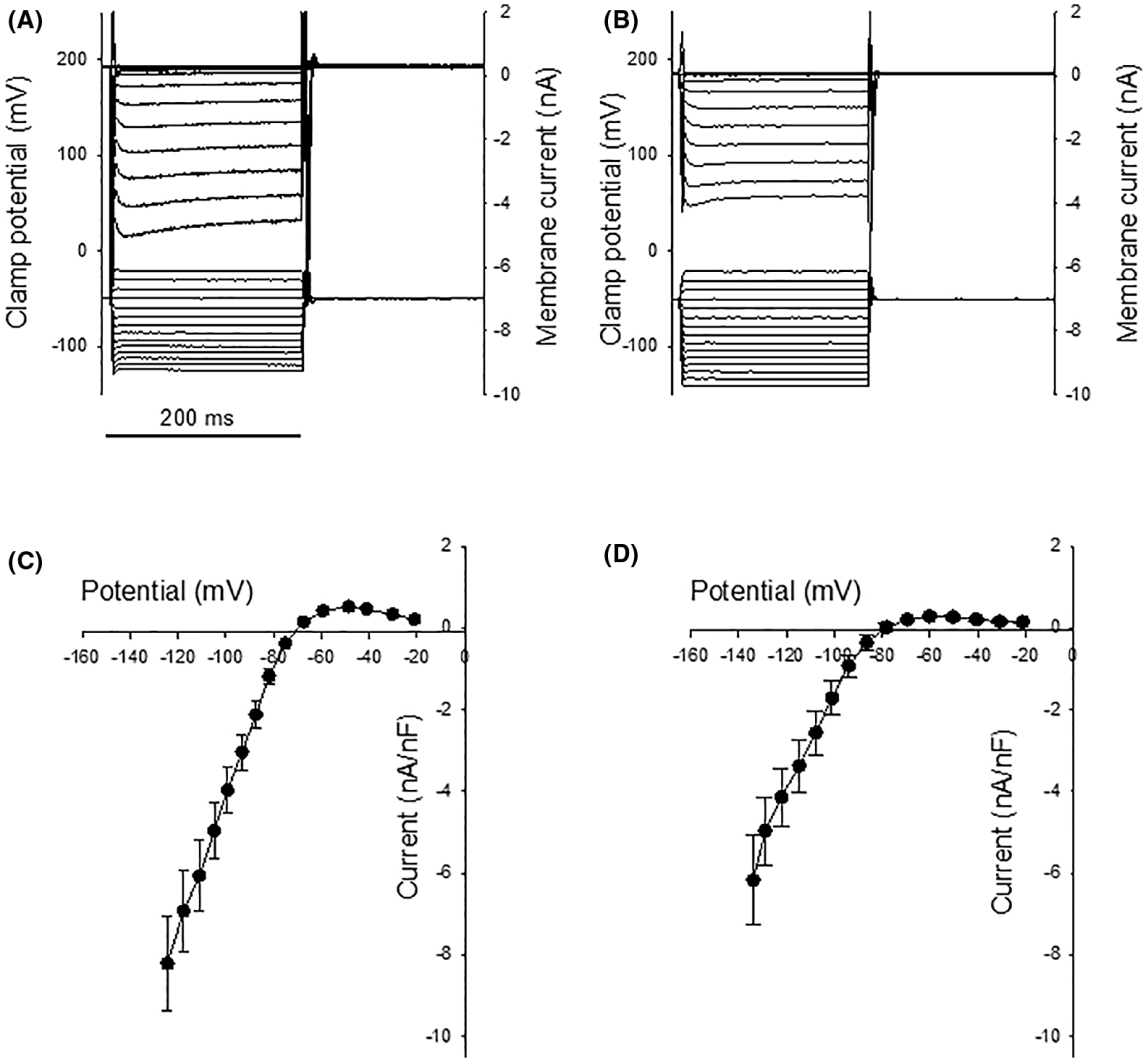
Original records of inward rectifier K^+^‐currents (I_K1_) determined in cardiomyocytes dissociated from a control heart (panel A) and a heart from a brain‐dead animal (panel B). The panels below show voltage–current relationships of control hearts (panel C, 25–29 cells from 9 animals) and hearts from brain‐dead animals (panel D, 11–13 cells from 5 animals). The I_K1_ was determined at the end of a 200 ms voltage step from a holding potential of −50 mV. Currents were normalized to the cell capacitance.

Figure 4 shows the properties of the inward rectifier K^+^ current (I_K1_) in isolated cardiomyocytes from controls and brain‐dead animals. The slope of the current voltage relationship in the conductance voltage range was less steep in the brain‐dead group compared to that of the controls (the slope of a linear regression for data points with voltage values less than – 80 mV was 113 ± 12, *n* = 28 pA/mV in the controls and 81 ± 12, *n* = 10 pA/mV in the brain‐dead group). When corrected for cell size, the corresponding values were 184 ± 25, *n* = 24 pA/(mV pF) in controls and 118 ± 21, *n* = 10 pA/(mV pF) in the brain‐dead group.

The maximal current in the rectifying voltage range above membrane potential was observed at lower (p < 0.001) membrane potential in the brain‐dead group (controls: −46.2 ± 1.6 mV, *n* = 25, brain dead: −57.2 ± 2.1 mV, *n* = 10). The maximal outward current was smaller (p < 0.05) in the brain‐dead group (controls: 530 ± 60 pA/pF, *n* = 25, brain dead: 300 ± 40 pA/pF, *n* = 10). The qPCR analysis showed a significantly lower mRNA for the Kir2.3 form of the IKr (KCNJ4), whereas the Kir2.1 form (KCNJ2) remained unchanged in the brain‐dead group (Table 2). Thus, a lower expression of Kir2.3 can be one contributing factor to the lower inward rectifying currents.

### 3.5 Transient outward (I_to_) and delayed rectifier (I_K_ ) K^+^‐currents

No transient outward currents could be detected consistent with previous reports,^17^, ^18^ showing that I_to_ K^+^ currents are less abundant or absent in the isolated pig cardiomyocytes. For evaluation of the delayed rectifier K^+^ current, we used a single step protocol (a 3 s step from −50 to +60 mV) in the presence of 5 μM Nifedipine. The average maximal I_K_ currents in response to the voltage step were similar in the two groups (controls: 0.73 ± 0.09 nA, *n* = 22 cells from 7 animals; brain dead: 0.73 ± 0.11 nA, *n* = 6 cells from 3 animals). An analysis of the time constant for the decay of the tail current following return to a holding potential of −25 mV after the voltage step showed a faster decay in the brain‐dead group (348.4 ± 52.1 ms, *n* = 28) compared to the control pig hearts (608.1 ± 95.4 ms, *n* = 19). Adding Chromanol decreased I_K_ by a similar proportion relative to the initial I_K_ in the two groups (controls: 29% ± 3%, *n* = 22; brain dead: 26% ± 5%, *n* = 6). Further addition of Dofetilide/E4031 resulted in an additional inhibition relative to the initial I_K_ (controls: 21% ± 3%, *n* = 5; brain dead: 3.4% ± 1.8%, *n* = 2). These results show that the delayed rectifier I_K_ is of similar amplitude in control and brain‐dead preparations. The I_Ks_ component is similar. I_Kr_ is possibly less prominent in the brain‐dead animals.

### 3.6 Intracellular Ca^2+^ transients

**FIGURE 5:**
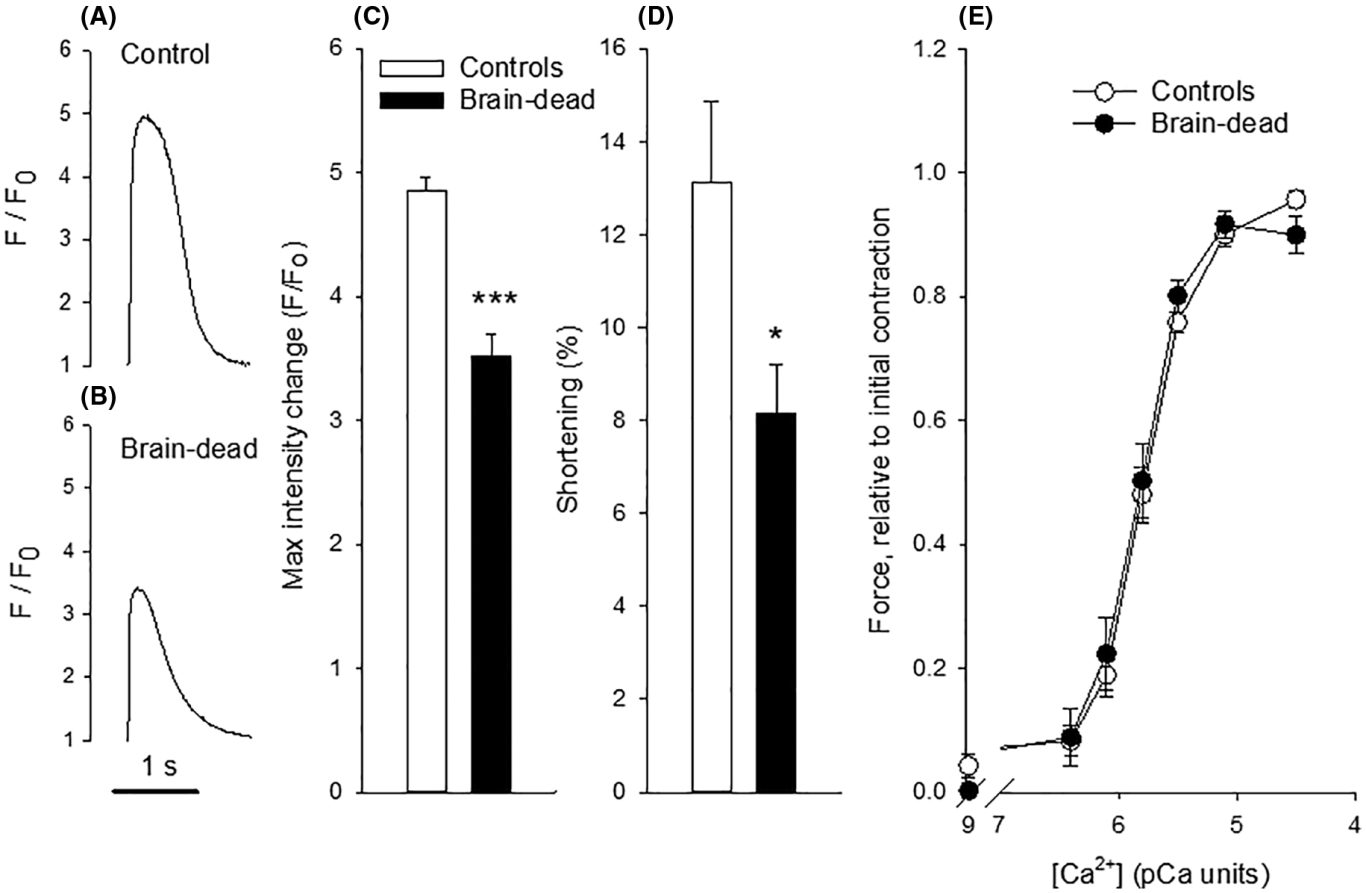
Fluorescence intensity change (F/F_0_) reflecting intracellular Ca^2+^‐transients (panel A and B), mean values of the Ca^2+^ changes (panel C) and shortening of isolated cardiomyocytes (panel D) from hearts of control (panel A) and brain‐dead animals (Panel B), *n* = 4 in each group. Panel E shows the active force at different free [Ca^2+^] determined in permeabilized trabecular preparations from these hearts. Force values are related to the force of an initial contraction at pCa 4.5.

Cardiomyocytes isolated from brain‐dead hearts showed a significantly lower fluorescence increase (F/F_0_) and shortening responses compared to cardiomyocytes from control hearts (Figure 5, panels A–D), suggesting an attenuated Ca^2+^ transient and impaired contractions. The qPCR analysis (Table 2) showed unaltered expression of the RyR2 receptor, of the SERCA2α subunit and of the sarcoplasmic reticulum ATPase and of the beta unit of the Na/K ATPase. However, the phospholamban expression was significantly lower.

### 3.7 Ca^2+^ sensitivity of the contractile system

Panel E of Figure 5 shows that the relationship between [Ca^2+^] and force of the permeabilized trabecular preparations was similar in control and brain‐dead groups. The EC_50_ values, that is, the pCa giving half maximal force, were calculated by fitting a hyperbolic equation. The EC_50_ values did not differ between the control and brain‐dead groups (control: 5.83 ± 0.03; brain dead 5.86 ± 0.06, pCa units, *n* = 4 in each group). The muscles relaxed completely between contractions as evident from the low initial tension at pCa 9. The force during the second activation at pCa 4.5 (i.e., the final step in the pCa‐force relationship) reached the same level as during the initial activation excluding instability in the force responses in either of the groups. Although Ca^2+^‐sensitivity can be accurately determined, we did not determine the maximal force of the skinned cardiac samples since these preparations are very thin and estimation of cross‐sectional area would introduce large errors.

### 3.8 Contraction of coronary arteries of different size

**FIGURE 6:**
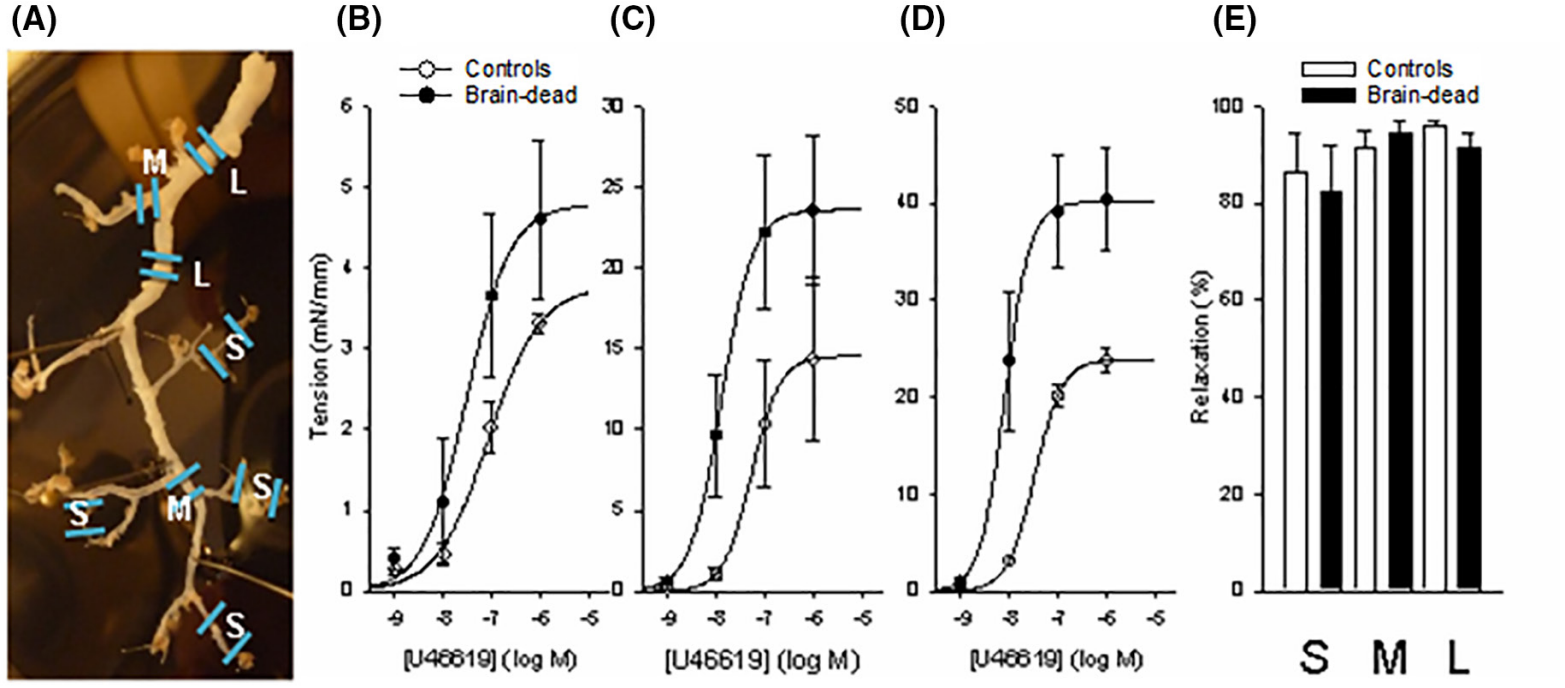
Panel A shows a photograph of dissected coronary artery branches with the location of the large (L, diameter ~ 2.5 mm), medium (M, ~1.4 mm), and small (S, 340 μm) arteries indicated. Panels B, C, and D show the contractile responses to the thromboxane A_2_ agonist (U46619) in S, M, and L arteries, respectively. Panel E shows percentage relaxation of the U46619 (1 μM) precontracted vessels in response to substance P (10 nM), in the different vessels, *n* = 4.

Mechanical properties, agonist induced contractions, and endothelial induced relaxations were examined in coronary arteries of three different diameters. The photograph in panel A of Figure 6 shows an isolated coronary artery tree with the different vessel segments indicated (large, medium, and small). Circumference‐tension relationships were determined for each preparation, and the corresponding diameters at optimal length (*L*_o_) are shown in Table 3. The diameters and passive tensions at *L*_o_ were similar in vessels of control and brain‐dead animals for the different vessel segments. The active tension (high‐K^+^ activation) at *L*_o_ was significantly increased in the large vessels of the brain‐dead group. The concentration‐tension relationship for the thromboxane A_2_‐receptor agonist U46619 was examined at *L*_o_ (Panel B–D, Figure 6) showing an increased maximal tension and an increased sensitivity for medium and larger vessels in the brain‐dead group. The relationships were analyzed by fitting the force (F) and concentration (C) data to a hyperbolic equation. The tension at saturating [U46619] was increased in the larger vessels of the brain‐dead group. The –log *EC*_*50*_ (the concentration giving half maximal response) values were higher in medium and larger vessels in the brain‐dead group (Table 3). These data show an increased contractility and increased sensitivity to U46619 in the coronary arteries of the brain‐dead pig heart. Endothelium mediated relaxation induced by substance P (10 nM) was examined in U46619 (1 μM) precontracted vessels. Relaxation was almost complete in all vessels, with no significant differences between the control and brain‐dead groups (Panel E, Figure 6). These results show that the substance P induced relaxation of the coronary arteries was not affected by the brain‐dead condition.

**TABLE 3:** Properties of coronary arteries. Mechanical properties of coronary arteries of different size from control and brain‐dead pigs (*n* = 4 in each group)

	Small		Medium		Large	
	Control	Brain dead	Control	Brain dead	Control	Brain dead
Diameter at *L*_o_ (μm)	343 ± 17	337 ± 36	1397 ± 162	1422 ± 184	2480 ± 132	2649 ± 160
Passive tension (mN/mm)	1.2 ± 0.2	0.9 ± 0.1	5.0 ± 1.0	5.3 ± 1.0	7.8 ± 2.1	8.6 ± 0.6
Active tension, high K^+^ (mN/mm)	4.4 ± 0.7	4.4 ± 0.9	12.8 ± 3.5	17.0 ± 2.7	17.4 ± 2.1	28.6 ± 3.8*
Max active tension, U46619 (mN/mm)	3.8 ± 0.2	4.7 ± 1.0	14.6 ± 5.1	23.5 ± 4.6	23.9 ± 1.2	40.3 ± 5.3*
EC_50_ for U46619 (−log M)	7.06 ± 0.24	7.45 ± 0.16	7.24 ± 0.04	7.81 ± 0.13**	7.48 ± 0.06	8.02 ± 0.16*

*Note*: Passive and maximal active tension (high‐K^+^ activation) at optimal length (*L*_o_) for active tension were determined in circumference‐tension experiments. The maximal active tension in response to U46619 was determined from the maximal value at saturating concentration when fitting a hyperbolic relation. * and ** indicate *p* < 0.05 and *p* < 0.01 compared to the control group.

## 4 DISCUSSION

We present data on membrane electrophysiology and Ca^2+^ handling of cardiomyocytes and on pharmacological reactivity of coronary arteries from hearts of brain‐dead pigs. A major conclusion is that the cardiac muscle and the cardiomyocytes preserve the main membrane ion channels during the 12 h brain‐dead period, but develop a shortened action potential, associated with impaired Ca^2+^ transients and contractile responses. Coronary arteries developed increased contractile responses and sensitivity to thromboxane stimulation, but had unchanged endothelial relaxant responses.

The pig model used in the present study^5^, ^12^ includes total removal of the brain and brainstem by decapitation, mimicking the effect of a rapid intracranial pressure increase with herniation of the brainstem in humans.^19^ Recent developments based on this pig model have enabled successful orthotopic transplantation of hearts harvested 24 h after brain death and preserved for 24 h.^5^ In this study, we applied a model with minimal interventions, resembling a serious clinical scenario, in order to identify key parameters that can be affected during a period with brain death. During the 12‐h observation period, minimal changes in blood gasses and acid base status were observed, excluding major problems in body perfusion. Both the hearts of the brain‐dead and of the control animals were treated in a similar manner, mimicking the clinical procedures prior to the transplantation. The hearts were thus excised from the animal and analyzed after a 4‐h period in cardioplegia.

To our knowledge, comparative data of cellular electrophysiological properties of brain‐dead versus fresh pig hearts have not been reported. In a previous study using surface electrodes on cardiac muscle during a 3 h brain‐dead period,^8^ it was found that the action potential or ischemia responses were not affected. Our studies include a longer brain‐dead period and a period in cardioplegia closer to the clinical situation, and it is possible that we identify changes in the myocardium that are not detectable on the epicardial surface or that develop at later stages, that is, after more than 3 h.

An important experimental aspect is that research on human cardiac tissue often involves comparisons of pathological cardiac tissue with samples from transplantation donor hearts, which might not constitute appropriate controls as pointed out by Jweied et al.^20^ Our results suggest that at least some electrophysiological properties of such “normal” or “control” hearts from brain‐dead subjects differ from those of fresh cardiac tissue.

It has been reported that post‐ischemic/stunned condition of the pig heart causes a decreased L‐type current density and attenuated Ca^2+^ transients.^21^ We find, both in trabecular and cellular preparations, a significantly shorter action potential in samples from the brain‐dead pigs compared to controls. The shortening responses and Ca^2+^ transients of isolated cardiomyocytes were significantly attenuated in the brain‐dead hearts. To obtain general information on membrane properties, and specifically explore underlying mechanisms for altered action potentials and shortening responses, we performed whole‐cell patch clamp and qPCR analyses of key ion currents/channel proteins. The general characteristics of the L‐type Ca^2+^ channels were similar in the control and brain‐dead groups, but both our electrophysiological and qPCR data suggest a lower activity in the channel. The peak of the Ca^2+^ transient, measured in the isolated cells, was reduced by almost 30% and it is possible that alterations in the Ca^2+^ storage of the sarcoplasmic reticulum, as suggested by the lower phospholamban expression are involved. We find that the expression of SERCA2 was slightly higher but not significantly changed. The lower phosoholamban expression, would, however, correlate with an increased uptake to sarcoplasmic reticulum and a shorter Ca^2+^ transient. The lower amplitude of the Ca^2+^‐transient cannot simply be explained by an increased pump activity, which would increase the loading of the sarcoplasmic reticulum. We could not detect a decrease in RYR2 receptors. It is thus likely that a decreased influx of Ca^2+^ via the Ca^2+^‐channels, as suggested by our electrophysiology and qPCR data, via impaired Ca^2+^ loading and attenuated Ca^2+^ induced Ca^2+^ release, contribute to a lower Ca^2+^ transient and impaired contractile response in the heart of the brain dead.

Cardiac ischemia has been shown to increase Ca^2+^ sensitivity at the myofilament level, possibly via proteolysis of troponin subunits.^22^, ^23^, ^24^ However, our studies of permeabilized trabecular preparations show that the myofilament Ca^2+^ sensitivity was not altered, excluding major changes in thin filament regulation. Additional modulation of Ca^2+^ sensitivity can be introduced by altered phosphorylation of regulatory proteins, including cardiac TnI and myosin binding protein C, both of which can be phosphorylated in the living heart under pathological conditions.^25^ We observe a clear correlation between altered Ca^2+^ transients and impaired contractility, which suggests that altered Ca^2+^ handling is a major change in the brain‐ dead heart. However, we cannot exclude additional changes in contractility and Ca^2+^ regulation with phosphorylation, possibly initiated by the catecholamine surge or hormonal changes.

K^+^ currents modulate the shape and duration of the action potential. In several species, an initial transient outward current (I_to_) contributes to initial repolarization after the upstroke phase. We show that this current is absent, or of very small amplitude, in the isolated pig cardiomyocytes (consistent with Refs. [17, 18]). The inward rectifier (I_K1_) had a slightly lower conductance and a significantly lower amplitude in the rectifying region, associated with a lower expression of the Kir2.2 channel form. A down regulation of this current is considered to lead to membrane instability and prolongation of the action potential.^26^, ^27^ It is therefore possible that changes in I_K1_ are associated with the increased risk of arrhythmias in a post‐transplantation period.^28^, ^29^ Alterations in this channel are, however, not simply responsible for the shorter action potential and attenuated Ca^2+^ transients observed in the present study, since lower K^+^ channel activity would prolong the action potential. We can also exclude major changes in the delayed rectifier (I_K_) currents.

Brain death involves several changes in the hemodynamics, nervous, and endocrine systems.^1^, ^2^ The factors triggering the change in Ca^2+^‐regulation are thus most likely complex. It has been shown that the plasma levels of thyroid hormone drop significantly during a period of brain death; T3 and T4 are decreased to about 40% and 50%, respectively, after 12 h in the pig brain‐dead model.^12^ Thyroid hormone is commonly used in clinical handling of brain death donors^4^, ^30^ to improve cardiac function. However, a drop in T3/T4 is most likely not responsible for the decrease in phospholamban and the small increase in SERCA pump expression, since hypothyroidism is well known to increase phospholamban and decrease SERCA.^31^ The L‐type calcium channel expression has been reported to be unaffected by hyperthyroidism.^32^ Also, the decrease in I_K1_ cannot simply be explained by hypothyroidism since thyroid hormone has been shown to decrease expression of this channel.^32^ It has also been shown that cortisol levels drop by more than 95% in brain‐dead animals.^12^ Glucocorticoids are found to increase the action potential duration, and the Ca^2+^‐channel influx^33^ which would be consistent with our data. Treatment with glucocorticoids has also been shown to be beneficial for cardiac function in a pig brain death model.^34^ However, glucocorticoids decrease phospholamban expression,^33^ which suggests that low glucocorticoids are not the sole explanation for our observations. Long‐term effects of catecholamine on pig heart ion channels and excitation‐contraction are not clear, and it is possible that the significant increase in both adrenaline and noradrenaline, about 80 and 300 times during the sympathetic storm or the subsequent drop to near undetectable levels^12^ can induce persistent changes.^35^ The low levels will affect blood pressure during the brain‐dead period and therapies have been directed to control noradrenaline levels by inhibiting neuronal uptake.^12^ In summary, several hormonal and physiological changes occurring during brain death might change expression key proteins in Ca^2+^ regulation. The drop in thyroid hormone seems to be less important in this context, but low cortisol and catecholamine levels might contribute.

The reactivity to the thromboxane A_2_ receptor agonist U46619 was dependent on the coronary artery size, with larger arteries more sensitive. After the brain‐dead period, the responses were significantly upregulated, an effect most pronounced in larger vessels. The reason for this alteration is unknown and can be associated with the prolonged anesthesia period, the hormonal changes or alterations in the neural input to the coronary arteries causing upregulation of the receptor responses, as discussed above. Intracellular changes in smooth muscle cannot be excluded, since responses to high‐K^+^ were also increased, but most likely the changes include alterations at the thromboxane receptor level. A functional consequence would be an increased risk of coronary spasm in the brain‐dead heart after transplantation. The endothelial relaxant responses were not affected, showing that the endothelial function was largely preserved during the brain‐dead period.

In conclusion, we report data on the myocardial cellular function after a period of brain death in a large animal model under conditions mimicking cardiac treatment during the clinical transplantation procedure. It should be noted that the present study was designed to identify critical issues applying a longer period of brain death without active interventions. Such hearts are in most cases excluded from transplantation, although information on their function is limited. We identified a shorter action potential and attenuated Ca^2+^ transients leading to impaired contractility. Minor alterations in the inward rectifier K^+^ current would not affect the contractility directly, but may be involved in an increased risk for cardiac arrhythmias.^27^ The sensitivity to thromboxane receptor activation and the contractile responses were increased in the coronary arteries of brain dead, possibly associated with an increased risk of impaired coronary perfusion. The time course and magnitude of the observed changes and the impact on the in vivo situation after transplantation as well as the possibility of restitution remain to be examined.

## AUTHOR CONTRIBUTIONS

Per Arlock, Mei Li, Stig Steen, and Anders Arner conceived and designed the study, performed experiments and data analyses, and wrote the manuscript. Benjamin Davis, Cecilia Lövdahl, Qiuming Liao, Trygve Sjöberg, Awahan Rahman, and Björn Wohlfart performed experiments and data analyses.

## DISCLOSURES

The authors declare no conflicts of interest.

## Data Availability

The data that support the findings of this study are available in the methods and/or results sections of this article.
